# Crystal structure of *p*-xylene@silicalite-1

**DOI:** 10.1107/S2056989024011642

**Published:** 2025-01-01

**Authors:** In-Chul Hwang

**Affiliations:** a School of Chemical and Biological Engineering, Seoul National University, 1, Gwanak-ro, Gwanak-gu, Seoul 08826, South Korea; University of Neuchâtel, Switzerland

**Keywords:** silicalite-1, *p*-xylene, zeolite, crystal structure

## Abstract

The crystal structure of a highly loaded complex of silicalite-1 (SL-1) with eight mol­ecules of *p*-xylene per unit cell has been solved by single-crystal X-ray diffraction. In the crystal, four symmetrical Si_24_O_48_·2C_8_H_10_ subunits per unit cell are observed. The *p*-xylene mol­ecules sit at two different positions within the SL-1 channels. The first mol­ecule is located at the inter­section of the sinusoidal and straight channels, while the second guest mol­ecule is positioned in the center of the double ten-membered ring (10-MR) of the sinusoidal channel.

## Abstract 2

In order to observe the maximum adsorption amount and arrangement state of *p*-xylene mol­ecules in a microporous single-crystal of silicalite-1 (SL-1), single-crystal X-ray diffraction analysis was performed.

## 1. Chemical context

Silicalite-1 (SL-1) has attracted considerable attention due to its wide applicability in shape-selective catalysts and adsorbents. Many aromatic sorbate-SL-1 structures have been investigated by single-crystal X-ray diffraction (van Koningsveld *et al.*, 1989 ▸; Reck *et al.*, 1996 ▸; van Koningsveld, Jansen & de Man, 1996 ▸; van Koningsveld, Jansen & van Bekkum, 1996 ▸; Nishi *et al.*, 2005 ▸; Kamiya *et al.*, 2011 ▸). In addition, Fujiyama and co-workers reported on the adsorption structures of various non-aromatic hydro­carbons on SL-1 using single-crystal X-ray diffraction (Fujiyama, Seino, Kamiya, Nishi, Yoza & Yokomori, 2014 ▸). They found that, depending on the guest–framework inter­actions, normal hydro­carbons prefer narrow channels, while the bulky iso­pentane prefers larger inter­sections. Additionally, they revealed that bent mol­ecules tend to prefer sinusoidal channels, whereas linear mol­ecules tend to favor straight channels.

Mixed xylenes (*p*-xylene, *m*-xylene, and *o*-xylene) are important chemical feedstocks used in the production of polyester fibers, resins, pigments, gasoline components, and more (Minceva *et al.*, 2008 ▸; Lusi & Barbour, 2012 ▸). Among these isomers, *p*-xylene has the highest application value and is a key raw material for the synthesis of refined terephthalic acid (PTA), polyethyl­ene terephthalate (PET), and other products (Torres-Knoop *et al.* 2014 ▸; Wu *et al.*. 2018 ▸; Ma *et al.* 2019 ▸).

The two main methods for obtaining high-purity *p*-xylene in industry are crystallization separation and simulated moving bed (SMB) adsorption separation technology (Mohameed *et al.*, 2007 ▸; Barcia *et al.*, 2012 ▸). Currently, research on the separation of *p*-xylene using porous zeolites has been ongoing (Caro-Ortiz *et al.*, 2021 ▸). van Koningsveld and co-workers reported the detailed structure of the ubiquitous adsorption sites of *p*-xylene mol­ecules in the channels of the microporous zeolite H-ZSM-5, Si_23.92_Al_0.08_O_48_·2(C_8_H_10_) (+0.08H^+^), and discussed the sorption mechanism of *p*-xylene mol­ecules in the channels (van Koningsveld *et al.*, 1989 ▸).

However, the *p*-xylene@SL-1 structure has not yet been determined by single-crystal X-ray diffraction. From this perspective, there is a need to closely examine the structure of *p*-xylene mol­ecules within zeolite channels, and this study was initiated. Single crystals of *p*-xylene@SL-1 were obtained by adsorbing *p*-xylene through vacuum and heat treatment.
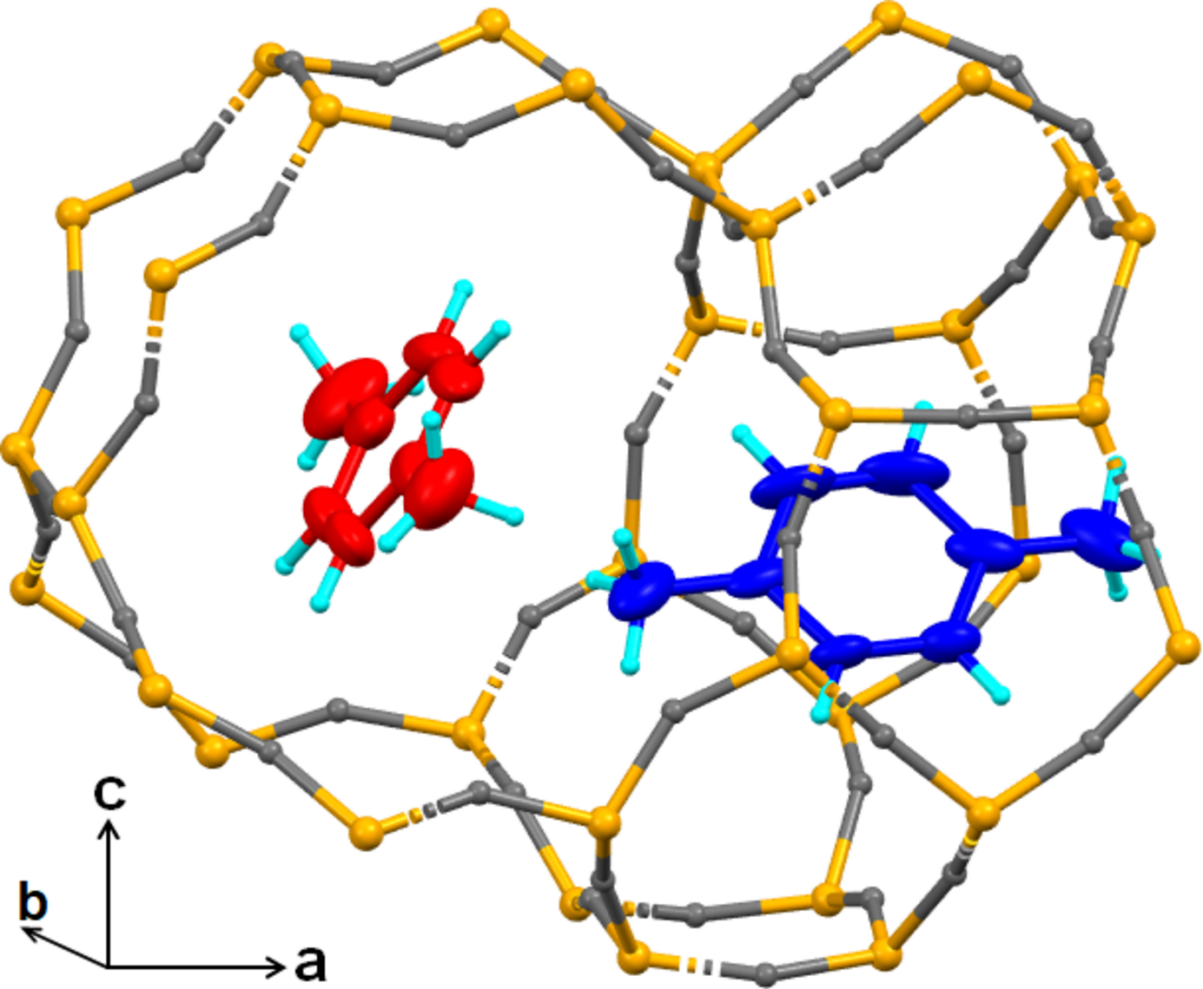


## 2. Structural commentary

**Figure 1:**
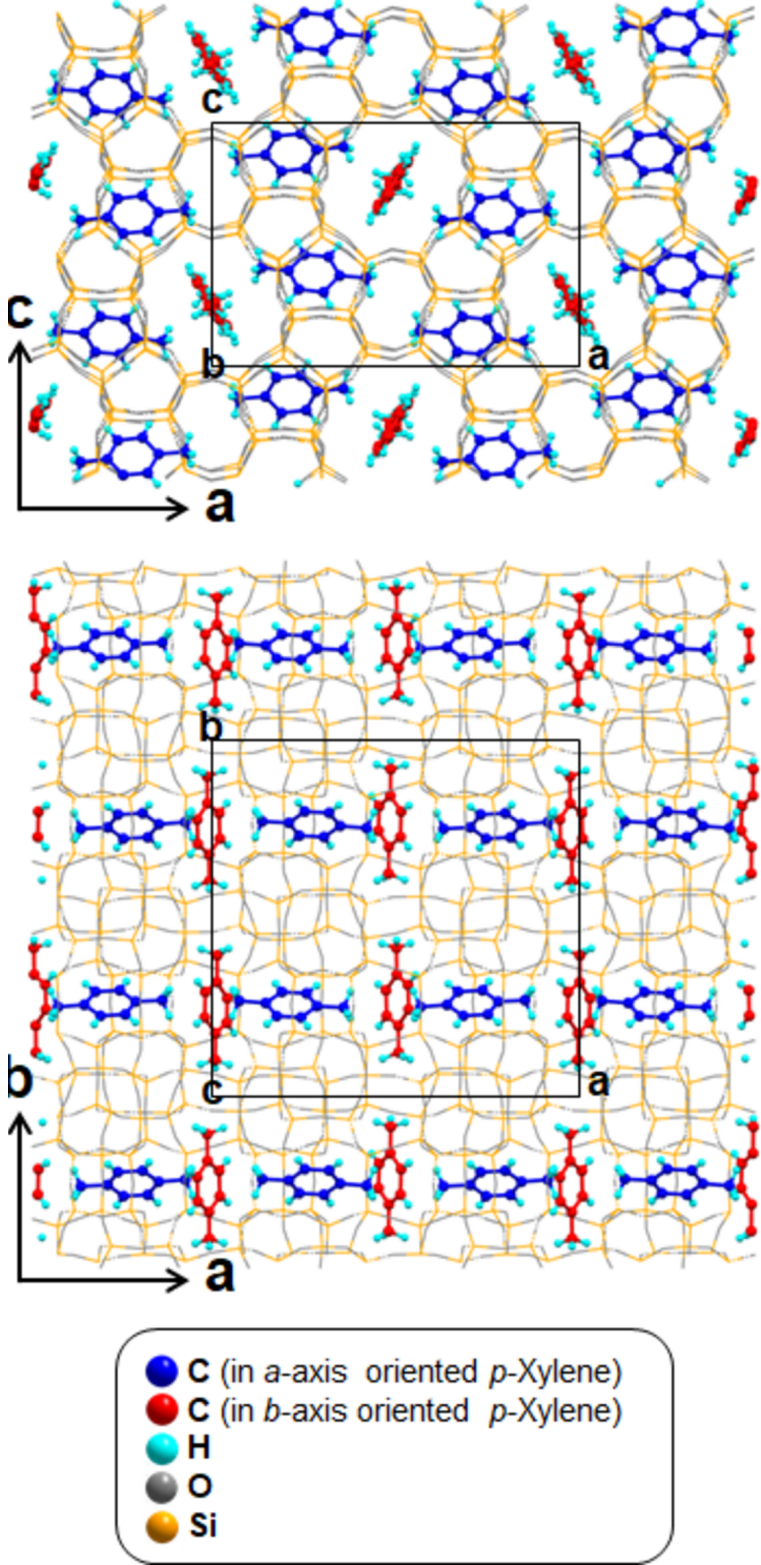
Unit-cell structures of *p*-xylene@SL-1 along the *b*-axis (top), and along the ***c***-axis (bottom). The carbon atoms of the *p*-xylene mol­ecules are drawn in two colors, the red-colored mol­ecules are located at the inter­section of the straight channel and sinusoidal channel, while the blue-colored mol­ecules are located in the sinusoidal channels.

The single-crystal structure of *p*-xylene@SL-1, (C_8_H_10_)_2_@Si_24_O_48_, has ortho­rhom­bic symmetry in space group *P*2_1_2_1_2_1_, whereas the original SL-1 framework has monoclinic symmetry in space group *P*2_1_/n (Pham *et al.*, 2016 ▸). Fig. 1 ▸ shows that *p*-xylene mol­ecules are arranged anisotropically in the channels of the SL-1 framework. One of the two independent *p*-xylene mol­ecules is located at the inter­section of the straight and sinusoidal channels, with its long mol­ecular axis nearly parallel to the straight channel axis. The second *p*-xylene mol­ecule lies in the sinusoidal channel, with its long mol­ecular axis nearly parallel to [100] (see Fig. 1 ▸). The minimal cross-section of the *p*-xylene mol­ecules fills the maximal limiting pores in both channels. The *p*-xylene mol­ecules in the sinusoidal channel are more tightly confined by the framework atoms than the mol­ecule in the straight channel. The main inter­action forces between the *p*-xylene mol­ecules at the inter­section of the channels and the one in the sinusoidal channel are almost identical to those in the [001] layer of *p*-xylene.

## 3. Supra­molecular features

The distances and angles of the SL-1 framework are summarized in the supporting information, along with the corresponding values for the *p*-xylene mol­ecules. The O—Si—O angles and Si—O distances are essentially similar to those reported in previous guest–silicalite-1 structures (van Koningsveld *et al.*, 1989 ▸; Fujiyama, Seino, Kamiya, Nishi, Yoza, & Yokomori, 2014 ▸).

**Figure 2:**
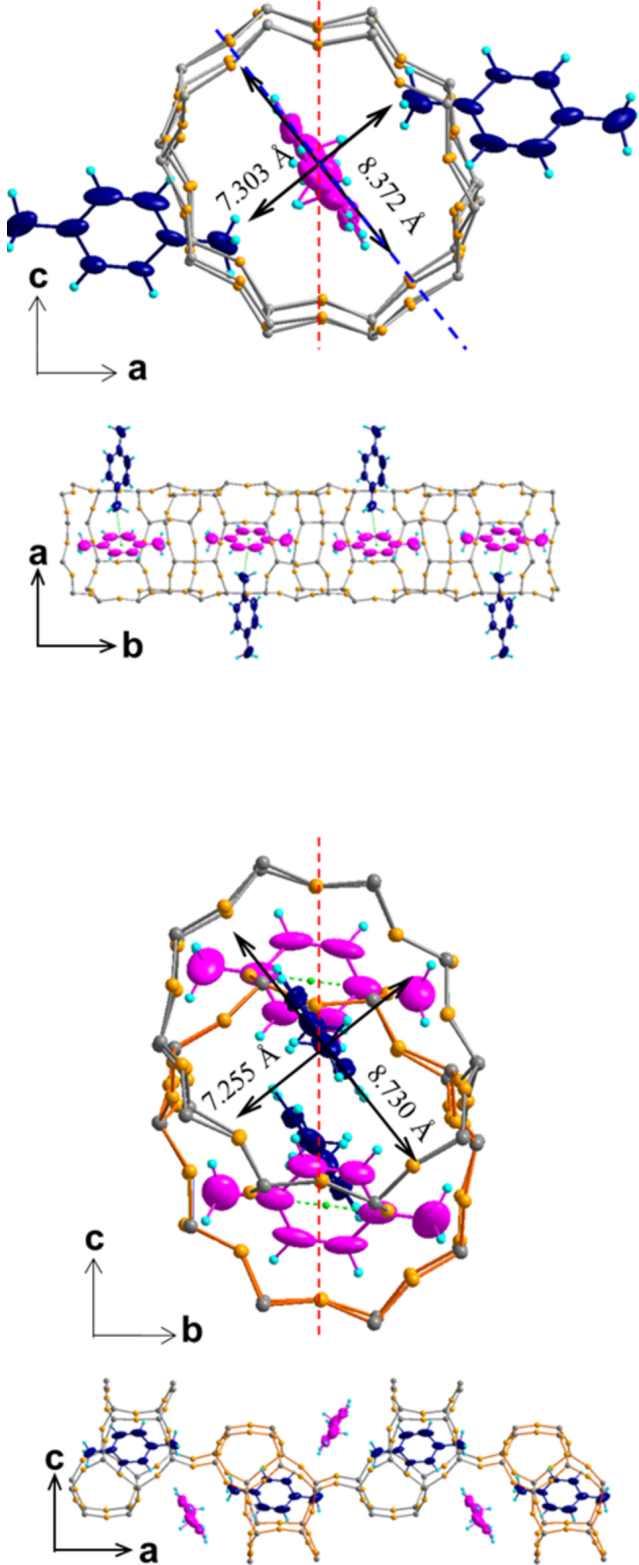
*p*-Xylene arrangement in the straight channel (top) and the sinusoidal channel (bottom), with carbon atoms shown as 50% probability displacement ellipsoids.

The elliptical ten-membered ring (10-MR) diameters of the straight and sinusoidal channels are 8.37 × 7.30 Å and 8.73 × 7.26 Å, respectively, corresponding to the diagonal distances between oxygen atoms along the major and minor axes. The straight channel, parallel to [010], is slightly corrugated, with elliptical cross-sections of 5.7 × 4.6 Å (r_oxygen_ = 1.35 Å), while the sinusoidal channel along [100] has dimensions of 6.0 × 4.6 Å (see Fig. 2 ▸). One *p*-xylene mol­ecule lies at the inter­section of the straight and sinusoidal channels, with its long axis nearly parallel to [100] and deviating by 7.45 (1)° from [010]. The angle between the benzene ring plane and the *a*-axis direction is −32.7 (1)°. The second *p*-xylene mol­ecule is in the sinusoidal channel, with its long axis deviating by 6.41 (1)° from [100] and nearly parallel to [010]. The angle between the benzene ring plane and the *b*-axis direction is −34.6 (1)° (see Fig. 2 ▸). The minimal cross sections of the *p*-xylene mol­ecules fill the maximal limiting pores in both channels.

Contacts between adjacent methyl groups of *p*-xylene mol­ecules in the straight channels are 4.18 (1) and 4.93 (1) Å, respectively. In the sinusoidal channel, the shortest CH_3_—CH_3_ distance between two C-atoms is 5.342 (1) Å, indicating that the packing forces between *p*-xylene mol­ecules are negligible.

**Figure 3:**
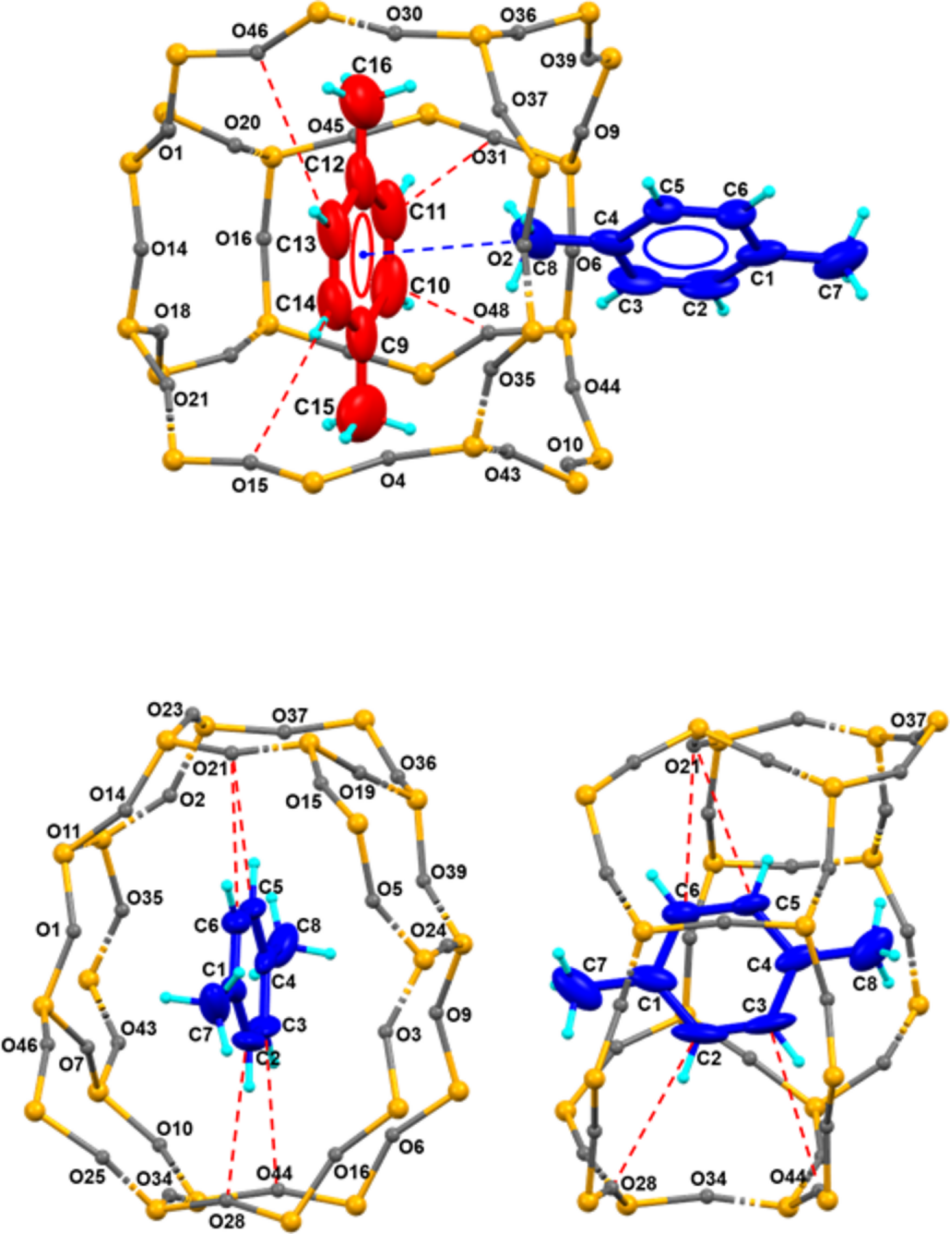
Illustrations of the inter­molecular inter­actions between *p*-xylene and oxygen atoms in SL-1. Straight channel (top) and sinusoidal channel (bottom) with arrangements of the *p*-xylene mol­ecules with carbon atoms shown as 50% probability displacement ellipsoids. The inter­molecular distance between independent *p*-xylene mol­ecules is *d*(C8⋯*Cg*) = 3.498 Å where *Cg* is the center of gravity of the C9–C14 ring.

Short *p*-xylene-to-SL-1 framework distances are summarized in Fig. 3 ▸ and Table 1 ▸, along with several short C—O contacts that may indicate (weak) H—O inter­actions. The *p*-xylene mol­ecule in the sinusoidal channel is more tightly packed (Fig. 3 ▸). On the other hand, the *p*-xylene mol­ecules at the channel inter­sections show contacts between a methyl group of one *p*-xylene with the center of the aromatic ring of an adjacent *p*-xylene with a distance of 3.50 (1) Å [see Fig. 3 ▸ (top)]. These structural features closely resemble those of the *p*-xylene/H-ZSM-5 complex (van Koningsveld *et al.*, 1989 ▸), except for the inter­action forces between the *p*-xylene mol­ecules at the channel inter­sections.

## 4. Database survey

A search of the Cambridge Structure Database (CSD, version 5.45, update June 2024; Groom *et al.*, 2016 ▸) gave several hits for small organic mol­ecules incorporated in SL-1. For example, Fujiyama and co-workers revealed that the adsorption structures of butane derivatives in SL-1 vary depending on the isomers, with bent mol­ecules preferring sinusoidal channels (NUVTIJ, *etc*.) and linear mol­ecules preferring straight channels (NUVQIG, *etc*.; Fujiyama, Seino, Kamiya, Nishi, Yoza, & Yokomori, 2014 ▸). Additionally, references to similar single-crystal structures incorporating small mol­ecules in the channels of SL-1 are as follows; CO_2_ (NUTHOB; Fujiyama, Kamiya, Nishi, & Yokomori, 2014 ▸), *n*-hexane (AHODOQ, AHODOQ01, AHODUW; Morell *et al.*, 2002 ▸), dimethyl ether (BOKLIY; Fujiyama, Seino, Kamiya, Nishi, & Yokomori, 2014 ▸), 1-butyl-3-methyl­imidazolium (FABNAZ; Wheatley *et al.*, 2010 ▸), toluol (FEWZUD; Nishi *et al.*, 2005 ▸), ethyl­enedi­amine (FIJYUT, FIKFEL; Perego *et al.*, 2003 ▸), tetra­propyl­ammonium hydroxide (FUHZUD; van Koningsveld *et al.*, 1987 ▸), pyridine (IQEBEM; Nishi *et al.*, 2007 ▸), phenyl (ODEVOK; Kamiya *et al.*, 2011 ▸), lithium hydroxide/tetra­propyl­ammonium hydroxide (PAGMIU; Park *et al.*, 2004 ▸), tetra­propyl­ammonium fluoride (PRAFSI10; Price *et al.*, 1982 ▸), methyl ether (QOTCIN, QOTCOT, QOTCUZ, QOTCUZ01; Fujiyama *et al.*, 2015 ▸), *para*-di­chloro­benzene (WEJJEA01; van Koningsveld, Jansen, & van Bekkum, 1996 ▸), and naphthalene (PUPPAR; van Koningsveld, & Jansen, 1996 ▸).

In summary, guest mol­ecules adsorbed in the microporous structure of SL-1, which consists of straight channels and sinusoidal channels, can be distinguished into three locations according to their shapes and sizes. The locations are the center of the double 10-MR in sinusoidal channels, the center of the double 10-MR in straight channels, and the inter­section between sinusoidal and straight channels.

## 5. Synthesis and crystallization

**Synthesis of SL-1:** Single crystals of pristine SL-1 with regular morphology were synthesized from a mixed gel (mole ratio; fumed silica: tetra­ethyl­ammonium hydroxide (TEAOH): KOH: NH_4_F: H_2_O = 1:0.48:0.1:0.18:15) under hydro­thermal reaction conditions (438 K, 12 days). SL-1 product was washed several times with distilled deionized water through sonication and centrifugation process. The washed pristine SL-1 was dried overnight at 353 K in a vacuum oven.

**Calcinations of pristine SL-1:** The zeolite calcinations process to remove the TEAOH organic template is quite important because the crystalline framework can be damaged depending on the temperature and time. In fact, since calcination above 823 K damages the crystal, two calcination periods of 12 h at 773 K, with a 2 h rest in between, have been performed.

**Preparation of*****p-*****xylene@SL-1:** In a glass vessel connected to a vacuum pump, SL-1 single-crystal (2.0 g) and *p*-xylene in a 5 ml glass vial were placed together, and heated in an oven under vacuum at 373 K for about 1 h. Then, when rapidly cooled to room temperature, *p*-xylene@SL-1 was obtained in which *p*-xylene mol­ecules were adsorbed in a saturated manner within the channels of SL-1.

## 6. Refinement

Crystal data, data collection and structure refinement details are summarized in Table 2 ▸. Synchrotron single-crystal X-ray diffraction data were obtained on an ADSC Quantum 210 CCD diffractometer at the Pohang Accelerator Laboratory (PAL) Large Mol­ecular Crystallography Wiggler two-dimensional beamline. The measured diffraction data were obtained using synchrotron radiation (*λ* = 0.7000 Å), and a total of 360 frames were measured by rotating the *ω* angle by 1.00° with an exposure time of 5 s per frame at 243 K. In the structure refinements, chemical elements were modified using the instruction of DISP (the dispersion and the absorption coefficient of particular elements) at the wavelength of 0.700 Å. The remaining hydrogen atoms were positioned geometrically and refined isotropically using a riding model, with C—H bond distances of 0.94 Å (phen­yl) and 0.97 Å (meth­yl) and *U*_iso_(H) = 1.2*U*_eq_(C) or 1.5*U*_eq_(C-meth­yl). The crystal studied was refined as a two-component inversion twin.

## Supplementary Material

Crystal structure: contains datablock(s) I. DOI: 10.1107/S2056989024011642/tx2091sup1.cif

Structure factors: contains datablock(s) I. DOI: 10.1107/S2056989024011642/tx2091Isup4.hkl

CCDC reference: 1477740

Additional supporting information:  crystallographic information; 3D view; checkCIF report

## Figures and Tables

**Table 1:** Selected interatomic distances (Å)

C1⋯O3	3.499 (1)	C6⋯O21^i^	3.319 (1)
C2⋯O28	3.489 (1)	C6⋯O1	3.439 (1)
C3⋯O6	3.326 (1)	C6⋯O14^i^	3.305 (1)
C3⋯O9	3.384 (1)	C10⋯O48^i^	3.830 (1)
C3⋯O44	3.411 (1)	C11⋯O31^i^	3.684 (1)
C4⋯O39	3.494 (1)	C11⋯O45^i^	3.684 (1)
C5⋯O39	3.428 (1)	C13⋯O46^i^	3.402 (1)
C5⋯O19^i^	3.440 (1)	C14⋯O15^i^	3.897 (1)
C5⋯O21^i^	3.315 (1)	C14⋯O21^i^	3.481 (1)
C5⋯O14^i^	3.151 (1)		

Symmetry code: (i) 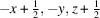.

**Table 2:** Experimental details

Crystal data	
Chemical formula	Si_24_O_48_·2C_8_H_10_
*M*_r_	1654.48
Crystal system, space group	Orthorhombic, *P*2_1_2_1_2_1_
Temperature (K)	243
*a*, *b*, *c* (Å)	20.128 (4), 19.816 (4), 13.429 (3)
*V* (Å^3^)	5356.2 (19)
*Z*	4
Radiation type	Synchrotron, λ = 0.700 Å
μ (mm^−1^)	0.65
Crystal size (mm)	0.02 × 0.02 × 0.01

Data collection	
Diffractometer	ADSC Quantum 210 CCD area detector
Absorption correction	Empirical (using intensity measurements) (*SCALEPACK*; Otwinowski & Minor, 1997 ▸)
*T*_min_, *T*_max_	0.987, 0.995
No. of measured, independent and observed [*I* > 2σ(*I*)] reflections	48262, 15347, 14416
*R*_int_	0.031
(sin θ/λ)_max_ (Å^−1^)	0.704

Refinement	
*R*[*F*^2^ > 2σ(*F*^2^)], *wR*(*F*^2^), *S*	0.028, 0.079, 1.04
No. of reflections	15347
No. of parameters	798
H-atom treatment	H-atom parameters constrained
Δρ_max_, Δρ_min_ (e Å^−3^)	0.60, −0.50
Absolute structure	Refined as a perfect inversion twin
Absolute structure parameter	0.5

Computer programs: PAL ADSC Quantum-210 ADX Program, *HKL-3000* (Otwinowski & Minor, 1997 ▸), *SHELXT2014/4* (Sheldrick, 2015*a* ▸), *SHELXL2019/2* (Sheldrick, 2015*b* ▸) and *OLEX2* (Dolomanov *et al.*, 2009 ▸; Bourhis *et al.*, 2015 ▸).

## supplementary crystallographic information

### p-Xylene@silicalite-1 Crystal data

**Table:**

Si_24_O_48_·2C_8_H_10_	*D*_x_ = 2.052 Mg m^−^^3^
*M**_r_* = 1654.48	Synchrotron radiation, λ = 0.700 Å
Orthorhombic, *P*2_1_2_1_2_1_	Cell parameters from 88940 reflections
*a* = 20.128 (4) Å	θ = 1.4–29.5°
*b* = 19.816 (4) Å	µ = 0.65 mm^−^^1^
*c* = 13.429 (3) Å	*T* = 243 K
*V* = 5356.2 (19) Å^3^	Stick, colorless
*Z* = 4	0.02 × 0.02 × 0.01 mm
*F*(000) = 3344	

### p-Xylene@silicalite-1 Data collection

**Table:**

ADSC Quantum 210 CCD area detector diffractometer	15347 independent reflections
Radiation source: PLSII 2D bending magnet	14416 reflections with *I* > 2σ(*I*)
Detector resolution: 4096 pixels mm^-1^	*R*_int_ = 0.031
ω–scan	θ_max_ = 29.5°, θ_min_ = 1.4°
Absorption correction: empirical (using intensity measurements) (Scalepack; Otwinowski & Minor, 1997)	*h* = −28→28
*T*_min_ = 0.987, *T*_max_ = 0.995	*k* = −27→27
48262 measured reflections	*l* = −18→18

### p-Xylene@silicalite-1 Refinement

**Table:**

Refinement on *F*^2^	H-atom parameters constrained
Least-squares matrix: full	*w* = 1/[σ^2^(*F*_o_^2^) + (0.0548*P*)^2^ + 0.467*P*] where *P* = (*F*_o_^2^ + 2*F*_c_^2^)/3
*R*[*F*^2^ > 2σ(*F*^2^)] = 0.028	(Δ/σ)_max_ = 0.002
*wR*(*F*^2^) = 0.079	Δρ_max_ = 0.60 e Å^−^^3^
*S* = 1.04	Δρ_min_ = −0.49 e Å^−^^3^
15347 reflections	Extinction correction: *SHELXL2019/2* (Sheldrick, 2015b), Fc^*^=kFc[1+0.001xFc^2^λ^3^/sin(2θ)]^-1/4^
798 parameters	Extinction coefficient: 0.0027 (2)
0 restraints	Absolute structure: Refined as a perfect inversion twin
Hydrogen site location: inferred from neighbouring sites	Absolute structure parameter: 0.5

### p-Xylene@silicalite-1 Special details

**Table:**

Geometry. All esds (except the esd in the dihedral angle between two l.s. planes) are estimated using the full covariance matrix. The cell esds are taken into account individually in the estimation of esds in distances, angles and torsion angles; correlations between esds in cell parameters are only used when they are defined by crystal symmetry. An approximate (isotropic) treatment of cell esds is used for estimating esds involving l.s. planes.
Refinement. The data-reduction process was defined as a unit cell and lattice system in the *HKL*-*3000* program, space group was determined by the *XPREP* program. The crystal structure of *p*-xylene@SL-1 was refined by direct methods using the *SHELXTL*-XS program and refined by full matrix least-squares calculation using *SHELXTL*-XL (ver. 2008) program package (Sheldrick, 2015) and Olex2 program (Dolomanov, *et al.*, 2009; Bourhis, *et al.*, 2015)). The crystal structure of p- xylene@SL-1 was solved in noncentrosymmetric orthorhombic space group P212121 (No. 19). All non-hydrogen atoms were refined anisotropically. Refined as a 2-component perfect inversion twin. 1. Fixed Uiso At 1.2 times of: All C(H) groups At 1.5 times of: All C(H,H,H) groups 2.a Aromatic/amide H refined with riding coordinates: C2(H2), C3(H3), C5(H5), C6(H6), C10(H10), C11(H11), C13(H13), C14(H14) 2.b Idealised Me refined as rotating group: C7(H7A,H7B,H7C), C8(H8A,H8B,H8C), C15(H15A,H15B,H15C), C16(H16A,H16B,H16C)

### p-Xylene@silicalite-1 Fractional atomic coordinates and isotropic or equivalent isotropic displacement parameters (Å^2^)

**Table:**

	*x*	*y*	*z*	*U*_iso_*/*U*_eq_	
C1	0.1480 (3)	0.2525 (2)	0.8689 (4)	0.0515 (13)	
C2	0.1916 (4)	0.2830 (2)	0.8012 (4)	0.0679 (17)	
H2	0.173981	0.306685	0.746655	0.081*	
C3	0.2595 (4)	0.2792 (2)	0.8122 (4)	0.0637 (16)	
H3	0.287027	0.300994	0.765684	0.076*	
C4	0.2885 (3)	0.24379 (19)	0.8905 (3)	0.0445 (11)	
C5	0.2452 (2)	0.2122 (2)	0.9561 (3)	0.0400 (9)	
H5	0.263149	0.186995	1.008937	0.048*	
C6	0.1777 (2)	0.2161 (2)	0.9472 (3)	0.0428 (10)	
H6	0.150566	0.194133	0.994057	0.051*	
C7	0.0741 (3)	0.2580 (3)	0.8608 (5)	0.074 (2)	
H7A	0.054240	0.214085	0.871807	0.112*	
H7B	0.062348	0.274101	0.794849	0.112*	
H7C	0.057817	0.289538	0.910383	0.112*	
C8	0.3614 (3)	0.2402 (3)	0.9014 (5)	0.0674 (17)	
H8A	0.379547	0.211139	0.849989	0.101*	
H8B	0.372363	0.221968	0.966359	0.101*	
H8C	0.380127	0.285060	0.894872	0.101*	
C9	0.5090 (3)	0.3324 (4)	0.7564 (5)	0.0683 (18)	
C10	0.4793 (3)	0.2873 (4)	0.6896 (4)	0.078 (2)	
H10	0.457548	0.304333	0.633000	0.093*	
C11	0.4812 (3)	0.2170 (4)	0.7051 (4)	0.077 (2)	
H11	0.459772	0.188567	0.659167	0.092*	
C12	0.5131 (3)	0.1890 (4)	0.7845 (5)	0.0707 (19)	
C13	0.5422 (3)	0.2342 (4)	0.8504 (5)	0.075 (2)	
H13	0.564150	0.217291	0.906864	0.091*	
C14	0.5400 (3)	0.3034 (4)	0.8360 (5)	0.0675 (17)	
H14	0.560704	0.331585	0.883055	0.081*	
C15	0.5074 (4)	0.4061 (5)	0.7403 (7)	0.112 (3)	
H15A	0.525917	0.428759	0.797989	0.168*	
H15B	0.461805	0.420591	0.730655	0.168*	
H15C	0.533357	0.417280	0.681791	0.168*	
C16	0.5138 (4)	0.1147 (4)	0.7958 (7)	0.100 (3)	
H16A	0.470857	0.099540	0.819852	0.149*	
H16B	0.547988	0.101908	0.843001	0.149*	
H16C	0.522933	0.093933	0.731806	0.149*	
O1	0.29938 (10)	0.64082 (8)	0.58803 (14)	0.0122 (3)	
O2	0.39007 (10)	1.24514 (9)	1.17883 (17)	0.0138 (4)	
O3	0.60073 (10)	0.35617 (9)	0.32381 (16)	0.0133 (4)	
O4	0.49742 (8)	−0.05418 (11)	0.47543 (16)	0.0147 (4)	
O5	0.40852 (10)	−0.07057 (10)	0.33702 (15)	0.0162 (4)	
O6	0.30167 (12)	0.25312 (9)	0.57585 (16)	0.0181 (4)	
O7	0.24416 (9)	0.56576 (10)	0.44914 (16)	0.0162 (4)	
O8	0.24224 (11)	0.33738 (12)	0.45610 (19)	0.0218 (5)	
O9	0.30155 (11)	0.14184 (8)	0.67630 (15)	0.0151 (4)	
O10	0.18159 (9)	0.54782 (10)	0.27877 (14)	0.0125 (3)	
O11	0.19512 (9)	0.65534 (9)	0.70339 (16)	0.0160 (4)	
O12	0.18492 (12)	0.45172 (10)	0.41565 (17)	0.0234 (4)	
O13	0.42386 (11)	0.04857 (10)	0.41406 (16)	0.0198 (4)	
O14	0.28217 (10)	0.75345 (9)	0.68150 (16)	0.0136 (4)	
O15	0.37364 (9)	−0.04866 (11)	0.52324 (16)	0.0177 (4)	
O16	0.57620 (12)	0.24562 (10)	0.42206 (18)	0.0208 (5)	
O17	0.60163 (12)	−0.00997 (11)	0.37934 (18)	0.0223 (5)	
O18	0.31129 (11)	0.83230 (10)	0.82970 (16)	0.0195 (4)	
O19	0.24416 (10)	−0.04377 (13)	0.51592 (17)	0.0230 (5)	
O20	0.11368 (10)	0.34204 (11)	0.44102 (17)	0.0171 (4)	
O21	0.30494 (11)	0.88039 (9)	0.64743 (16)	0.0176 (4)	
O22	0.41691 (11)	0.16696 (11)	0.33076 (18)	0.0220 (5)	
O23	0.19685 (10)	0.84536 (10)	0.7406 (2)	0.0253 (5)	
O24	0.19998 (10)	0.06581 (11)	0.73077 (19)	0.0255 (5)	
O25	0.41272 (11)	0.55262 (12)	0.28872 (16)	0.0214 (4)	
O26	0.09152 (11)	0.01283 (10)	0.66209 (18)	0.0219 (5)	
O27	0.38698 (10)	0.85426 (12)	0.98099 (17)	0.0185 (4)	
O28	0.60159 (11)	0.12256 (10)	0.37160 (18)	0.0200 (4)	
O29	0.25891 (12)	0.85280 (14)	1.0056 (2)	0.0290 (6)	
O30	0.50287 (8)	1.04870 (12)	1.06371 (17)	0.0184 (4)	
O31	0.37004 (11)	0.14891 (12)	0.51168 (19)	0.0230 (5)	
O32	0.41187 (11)	0.44994 (11)	0.41273 (19)	0.0257 (5)	
O33	0.31349 (13)	0.95108 (10)	0.90863 (18)	0.0278 (5)	
O34	0.70180 (9)	0.05429 (12)	0.30234 (18)	0.0264 (5)	
O35	0.41308 (10)	0.36085 (9)	0.09386 (16)	0.0131 (4)	
O36	0.38387 (9)	1.04763 (14)	0.99205 (17)	0.0224 (5)	
O37	0.41130 (13)	1.11865 (11)	1.1497 (2)	0.0303 (6)	
O38	0.31293 (10)	0.64401 (10)	0.78159 (15)	0.0139 (4)	
O39	0.30783 (10)	1.07199 (9)	0.83972 (14)	0.0127 (3)	
O40	0.49670 (9)	0.35045 (12)	0.44219 (18)	0.0183 (4)	
O41	0.42159 (11)	0.35132 (12)	0.28593 (17)	0.0205 (4)	
O42	0.29762 (12)	0.51056 (10)	0.60757 (16)	0.0212 (4)	
O43	0.11377 (9)	0.56067 (10)	0.44167 (15)	0.0136 (4)	
O44	0.30331 (12)	0.38034 (10)	0.61749 (16)	0.0213 (4)	
O45	0.49648 (9)	0.14852 (11)	0.48007 (17)	0.0176 (4)	
O46	0.37296 (9)	0.56810 (10)	0.47314 (16)	0.0159 (4)	
O47	0.30872 (11)	0.01096 (9)	0.66601 (16)	0.0162 (4)	
O48	0.37061 (10)	0.33190 (11)	0.4650 (2)	0.0230 (5)	
Si1	0.42550 (3)	−0.03090 (3)	0.43722 (5)	0.00494 (12)	
Si2	0.31735 (3)	0.87229 (3)	0.93152 (5)	0.00586 (12)	
Si3	0.18148 (3)	0.53140 (3)	0.39587 (5)	0.00567 (12)	
Si4	0.62335 (3)	0.05474 (3)	0.31636 (5)	0.00589 (12)	
Si5	0.30290 (3)	0.57070 (3)	0.52909 (5)	0.00484 (11)	
Si6	0.30393 (3)	0.17308 (3)	0.56638 (5)	0.00556 (11)	
Si7	0.38007 (3)	1.17145 (3)	1.22503 (6)	0.00657 (13)	
Si8	0.27894 (3)	0.07270 (3)	0.72846 (5)	0.00558 (12)	
Si9	0.30721 (3)	−0.04994 (3)	0.58746 (5)	0.00548 (11)	
Si10	0.31486 (3)	1.02889 (3)	0.93970 (5)	0.00639 (12)	
Si11	0.38195 (3)	0.32528 (3)	0.19052 (6)	0.00612 (12)	
Si12	0.42542 (3)	0.37125 (3)	0.40143 (5)	0.00596 (12)	
Si13	0.12141 (3)	0.07337 (3)	0.72653 (5)	0.00523 (12)	
Si14	0.18189 (3)	0.37195 (3)	0.39818 (5)	0.00600 (12)	
Si15	0.57203 (3)	0.16915 (3)	0.45858 (5)	0.00624 (12)	
Si16	0.27250 (3)	0.67380 (3)	0.68885 (5)	0.00513 (12)	
Si17	0.42577 (3)	1.05029 (3)	1.09251 (5)	0.00547 (11)	
Si18	0.42620 (3)	0.12801 (3)	0.43403 (5)	0.00593 (12)	
Si19	0.30412 (3)	0.32698 (3)	0.52895 (5)	0.00546 (12)	
Si20	0.42370 (3)	0.52956 (3)	0.40213 (5)	0.00565 (12)	
Si21	0.57473 (3)	−0.06593 (3)	0.45433 (5)	0.00522 (12)	
Si22	0.27977 (3)	0.44711 (3)	0.67508 (5)	0.00592 (12)	
Si23	0.57171 (3)	0.32600 (3)	0.42638 (5)	0.00544 (12)	
Si24	0.27405 (3)	0.82808 (3)	0.72431 (5)	0.00614 (13)	

### p-Xylene@silicalite-1 Atomic displacement parameters (Å^2^)

**Table:**

	*U*^11^	*U*^22^	*U*^33^	*U*^12^	*U*^13^	*U*^23^
C1	0.096 (4)	0.0269 (18)	0.031 (3)	0.012 (2)	−0.010 (3)	−0.0031 (16)
C2	0.136 (6)	0.034 (2)	0.033 (3)	0.011 (3)	−0.005 (3)	0.0147 (18)
C3	0.124 (5)	0.034 (2)	0.033 (3)	−0.007 (3)	0.020 (3)	0.015 (2)
C4	0.081 (3)	0.0265 (17)	0.026 (2)	−0.0090 (19)	0.018 (2)	−0.0064 (14)
C5	0.065 (3)	0.0323 (19)	0.022 (2)	0.0010 (18)	0.0105 (19)	0.0065 (15)
C6	0.064 (3)	0.0345 (19)	0.030 (2)	0.0040 (18)	0.0051 (19)	0.0058 (16)
C7	0.102 (5)	0.060 (3)	0.062 (4)	0.035 (3)	−0.032 (4)	−0.022 (3)
C8	0.081 (4)	0.068 (3)	0.054 (4)	−0.023 (3)	0.027 (3)	−0.020 (3)
C9	0.039 (3)	0.121 (6)	0.045 (3)	−0.004 (3)	0.012 (2)	0.004 (3)
C10	0.052 (3)	0.152 (7)	0.029 (3)	0.009 (4)	0.001 (2)	0.004 (4)
C11	0.050 (3)	0.145 (7)	0.035 (3)	−0.014 (4)	0.006 (2)	−0.029 (4)
C12	0.038 (2)	0.127 (6)	0.048 (4)	−0.015 (3)	0.009 (2)	−0.017 (4)
C13	0.041 (3)	0.136 (7)	0.049 (4)	−0.013 (3)	−0.012 (2)	−0.009 (4)
C14	0.043 (3)	0.107 (5)	0.052 (4)	−0.010 (3)	−0.009 (2)	−0.013 (3)
C15	0.105 (6)	0.126 (7)	0.104 (7)	0.014 (5)	0.055 (6)	0.001 (6)
C16	0.082 (5)	0.112 (7)	0.104 (7)	−0.014 (4)	0.032 (5)	0.000 (5)
O1	0.0207 (9)	0.0081 (7)	0.0078 (9)	0.0018 (7)	0.0022 (8)	−0.0039 (6)
O2	0.0215 (9)	0.0052 (7)	0.0146 (11)	0.0003 (6)	0.0013 (8)	0.0032 (7)
O3	0.0216 (9)	0.0084 (8)	0.0098 (10)	−0.0009 (7)	0.0038 (8)	0.0037 (7)
O4	0.0060 (7)	0.0241 (10)	0.0142 (11)	0.0053 (7)	−0.0031 (6)	−0.0036 (8)
O5	0.0280 (10)	0.0141 (9)	0.0066 (10)	0.0004 (7)	−0.0073 (8)	−0.0028 (7)
O6	0.0339 (11)	0.0061 (7)	0.0144 (11)	0.0020 (8)	0.0091 (10)	0.0037 (6)
O7	0.0137 (8)	0.0213 (10)	0.0137 (11)	−0.0043 (7)	−0.0054 (7)	−0.0003 (8)
O8	0.0166 (9)	0.0268 (11)	0.0220 (13)	0.0046 (8)	−0.0107 (9)	0.0015 (9)
O9	0.0296 (10)	0.0081 (7)	0.0077 (9)	−0.0042 (7)	0.0001 (8)	0.0038 (6)
O10	0.0108 (7)	0.0228 (9)	0.0039 (8)	−0.0005 (7)	0.0022 (6)	−0.0004 (7)
O11	0.0073 (8)	0.0151 (8)	0.0255 (11)	0.0002 (7)	−0.0018 (8)	0.0007 (7)
O12	0.0424 (12)	0.0077 (8)	0.0202 (11)	0.0000 (9)	0.0045 (9)	−0.0017 (8)
O13	0.0309 (10)	0.0076 (8)	0.0210 (11)	0.0006 (8)	−0.0018 (8)	−0.0009 (8)
O14	0.0235 (9)	0.0045 (7)	0.0129 (11)	−0.0016 (6)	0.0019 (8)	−0.0032 (6)
O15	0.0109 (8)	0.0267 (10)	0.0154 (11)	0.0013 (8)	0.0087 (7)	0.0036 (9)
O16	0.0359 (11)	0.0057 (8)	0.0208 (13)	0.0004 (8)	0.0063 (10)	0.0026 (7)
O17	0.0356 (12)	0.0140 (9)	0.0173 (12)	−0.0053 (8)	0.0054 (10)	0.0056 (8)
O18	0.0257 (10)	0.0228 (9)	0.0101 (10)	0.0052 (8)	−0.0086 (9)	−0.0073 (7)
O19	0.0154 (9)	0.0377 (13)	0.0160 (11)	0.0035 (9)	−0.0091 (8)	−0.0035 (10)
O20	0.0104 (8)	0.0269 (11)	0.0141 (11)	−0.0047 (7)	0.0060 (8)	−0.0012 (8)
O21	0.0266 (10)	0.0074 (7)	0.0187 (11)	−0.0030 (8)	0.0047 (9)	0.0059 (7)
O22	0.0274 (11)	0.0216 (10)	0.0171 (12)	−0.0065 (8)	−0.0130 (9)	0.0106 (8)
O23	0.0072 (8)	0.0250 (10)	0.0437 (15)	−0.0016 (8)	0.0017 (10)	−0.0161 (9)
O24	0.0064 (8)	0.0358 (12)	0.0342 (13)	0.0020 (8)	0.0011 (9)	0.0087 (10)
O25	0.0257 (10)	0.0303 (11)	0.0081 (10)	−0.0010 (9)	−0.0070 (8)	0.0029 (9)
O26	0.0336 (12)	0.0135 (9)	0.0186 (12)	−0.0028 (8)	−0.0079 (9)	−0.0089 (8)
O27	0.0115 (9)	0.0332 (12)	0.0110 (11)	0.0055 (8)	−0.0050 (8)	0.0021 (9)
O28	0.0295 (11)	0.0120 (9)	0.0184 (12)	0.0036 (8)	0.0047 (9)	−0.0065 (8)
O29	0.0197 (11)	0.0470 (15)	0.0202 (13)	−0.0070 (10)	0.0106 (9)	0.0044 (11)
O30	0.0046 (7)	0.0352 (11)	0.0153 (11)	0.0016 (8)	−0.0002 (7)	0.0044 (10)
O31	0.0147 (10)	0.0277 (11)	0.0267 (13)	0.0049 (8)	0.0105 (9)	−0.0037 (9)
O32	0.0336 (11)	0.0081 (8)	0.0355 (14)	0.0024 (8)	0.0028 (10)	−0.0011 (9)
O33	0.0506 (14)	0.0082 (8)	0.0245 (12)	0.0050 (10)	−0.0032 (11)	0.0029 (8)
O34	0.0062 (8)	0.0403 (12)	0.0327 (13)	0.0020 (9)	0.0028 (8)	0.0020 (10)
O35	0.0193 (9)	0.0080 (8)	0.0121 (10)	−0.0015 (6)	0.0049 (7)	0.0039 (7)
O36	0.0098 (8)	0.0452 (13)	0.0123 (11)	−0.0067 (9)	−0.0063 (7)	0.0069 (10)
O37	0.0485 (15)	0.0109 (9)	0.0315 (15)	0.0051 (9)	0.0160 (12)	−0.0066 (9)
O38	0.0142 (8)	0.0195 (9)	0.0080 (9)	0.0027 (7)	−0.0030 (7)	0.0031 (7)
O39	0.0213 (9)	0.0108 (7)	0.0060 (9)	−0.0002 (7)	−0.0039 (8)	0.0028 (6)
O40	0.0071 (8)	0.0344 (11)	0.0133 (11)	0.0055 (7)	−0.0033 (7)	0.0020 (9)
O41	0.0185 (10)	0.0321 (11)	0.0109 (11)	−0.0015 (8)	−0.0048 (8)	−0.0069 (9)
O42	0.0368 (12)	0.0118 (8)	0.0151 (11)	−0.0042 (8)	−0.0014 (10)	0.0068 (7)
O43	0.0091 (7)	0.0236 (10)	0.0081 (10)	0.0037 (7)	0.0049 (7)	−0.0014 (8)
O44	0.0369 (12)	0.0116 (8)	0.0154 (11)	0.0055 (9)	−0.0050 (10)	−0.0058 (7)
O45	0.0092 (9)	0.0290 (11)	0.0146 (11)	−0.0061 (7)	−0.0037 (7)	0.0016 (9)
O46	0.0090 (8)	0.0187 (9)	0.0201 (11)	0.0006 (7)	0.0068 (7)	−0.0089 (8)
O47	0.0229 (9)	0.0098 (8)	0.0158 (10)	0.0031 (7)	0.0003 (9)	−0.0062 (7)
O48	0.0146 (9)	0.0226 (10)	0.0320 (14)	−0.0010 (8)	0.0121 (9)	0.0070 (9)
Si1	0.0042 (2)	0.0063 (3)	0.0043 (3)	0.00079 (19)	−0.0002 (2)	−0.0009 (2)
Si2	0.0066 (3)	0.0059 (2)	0.0051 (3)	0.0004 (2)	−0.0010 (2)	0.0004 (2)
Si3	0.0064 (3)	0.0068 (3)	0.0039 (3)	−0.0003 (2)	0.0012 (2)	−0.0013 (2)
Si4	0.0060 (2)	0.0065 (3)	0.0052 (3)	−0.0001 (2)	0.0006 (2)	−0.0008 (2)
Si5	0.0059 (2)	0.0047 (2)	0.0039 (3)	0.0004 (2)	0.0008 (2)	−0.00106 (19)
Si6	0.0067 (2)	0.0047 (2)	0.0053 (3)	0.0001 (2)	0.0003 (2)	0.0009 (2)
Si7	0.0077 (3)	0.0035 (2)	0.0085 (4)	0.0014 (2)	−0.0007 (2)	0.0017 (2)
Si8	0.0065 (3)	0.0056 (3)	0.0047 (3)	0.0008 (2)	−0.0004 (2)	0.0019 (2)
Si9	0.0060 (2)	0.0058 (2)	0.0046 (3)	−0.0003 (2)	0.0016 (2)	0.0005 (2)
Si10	0.0061 (3)	0.0078 (3)	0.0054 (3)	0.0007 (2)	−0.0011 (2)	0.0021 (2)
Si11	0.0067 (3)	0.0051 (3)	0.0066 (3)	−0.0008 (2)	−0.0003 (2)	0.0005 (2)
Si12	0.0040 (3)	0.0060 (3)	0.0078 (3)	0.0003 (2)	−0.0001 (2)	0.0002 (2)
Si13	0.0056 (2)	0.0063 (2)	0.0038 (3)	−0.0003 (2)	−0.0001 (2)	0.0010 (2)
Si14	0.0060 (3)	0.0062 (3)	0.0058 (3)	0.0006 (2)	0.0019 (2)	0.0001 (2)
Si15	0.0063 (3)	0.0043 (3)	0.0082 (3)	0.0003 (2)	−0.0017 (2)	0.0001 (2)
Si16	0.0068 (3)	0.0036 (2)	0.0049 (3)	0.0011 (2)	−0.0007 (2)	−0.0013 (2)
Si17	0.0044 (2)	0.0061 (2)	0.0059 (3)	0.0006 (2)	0.0004 (2)	0.0005 (2)
Si18	0.0046 (3)	0.0058 (3)	0.0074 (3)	−0.0001 (2)	−0.0010 (2)	0.0013 (2)
Si19	0.0065 (2)	0.0035 (2)	0.0063 (3)	0.0006 (2)	0.0013 (2)	0.0015 (2)
Si20	0.0041 (2)	0.0070 (3)	0.0058 (3)	0.0008 (2)	0.0000 (2)	−0.0012 (2)
Si21	0.0048 (2)	0.0056 (3)	0.0053 (3)	0.00036 (19)	−0.0008 (2)	−0.0015 (2)
Si22	0.0073 (2)	0.0053 (2)	0.0052 (3)	−0.0004 (2)	−0.0019 (2)	−0.0003 (2)
Si23	0.0053 (2)	0.0045 (2)	0.0065 (3)	−0.0002 (2)	−0.0012 (2)	0.0015 (2)
Si24	0.0080 (3)	0.0041 (2)	0.0063 (3)	−0.0013 (2)	−0.0002 (2)	−0.0015 (2)

### p-Xylene@silicalite-1 Geometric parameters (Å, º)

**Table:**

C1—C2	1.400 (8)	O16—Si15	1.595 (2)
C1—C6	1.408 (6)	O16—Si23	1.597 (2)
C1—C7	1.495 (8)	O17—Si4	1.597 (2)
C2—H2	0.9400	O17—Si21	1.593 (2)
C2—C3	1.378 (9)	O18—Si2	1.585 (2)
C3—H3	0.9400	O18—Si24	1.604 (2)
C3—C4	1.391 (7)	O19—Si9	1.596 (2)
C4—C5	1.387 (6)	O19—Si10^iv^	1.596 (2)
C4—C8	1.477 (8)	O20—Si14	1.602 (2)
C5—H5	0.9400	O20—Si15^vi^	1.603 (2)
C5—C6	1.367 (6)	O21—Si9^vii^	1.5990 (19)
C6—H6	0.9400	O21—Si24	1.590 (2)
C7—H7A	0.9700	O22—Si7^viii^	1.604 (2)
C7—H7B	0.9700	O22—Si18	1.598 (2)
C7—H7C	0.9700	O23—Si7^ix^	1.597 (2)
C8—H8A	0.9700	O23—Si24	1.606 (2)
C8—H8B	0.9700	O24—Si8	1.595 (2)
C8—H8C	0.9700	O24—Si13	1.590 (2)
C9—C10	1.400 (9)	O25—Si4^x^	1.587 (2)
C9—C14	1.365 (9)	O25—Si20	1.605 (2)
C9—C15	1.476 (10)	O26—Si13	1.597 (2)
C10—H10	0.9400	O26—Si17^iv^	1.599 (2)
C10—C11	1.408 (10)	O27—Si2	1.592 (2)
C11—H11	0.9400	O27—Si23^xi^	1.598 (2)
C11—C12	1.364 (9)	O28—Si4	1.596 (2)
C12—C13	1.390 (8)	O28—Si15	1.603 (2)
C12—C16	1.479 (10)	O29—Si2	1.588 (2)
C13—H13	0.9400	O29—Si6^v^	1.590 (2)
C13—C14	1.385 (9)	O30—Si17	1.5997 (18)
C14—H14	0.9400	O30—Si20^xi^	1.5934 (19)
C15—H15A	0.9700	O31—Si6	1.594 (2)
C15—H15B	0.9700	O31—Si18	1.593 (2)
C15—H15C	0.9700	O32—Si12	1.590 (2)
C16—H16A	0.9700	O32—Si20	1.602 (2)
C16—H16B	0.9700	O33—Si2	1.593 (2)
C16—H16C	0.9700	O33—Si10	1.597 (2)
O1—Si5	1.6007 (18)	O34—Si4	1.590 (2)
O1—Si16	1.598 (2)	O34—Si22^ii^	1.599 (2)
O2—Si7	1.5993 (19)	O35—Si11	1.604 (2)
O2—Si11^i^	1.6042 (19)	O35—Si21^x^	1.6075 (19)
O3—Si13^ii^	1.6061 (19)	O36—Si10	1.601 (2)
O3—Si23	1.611 (2)	O36—Si17	1.592 (2)
O4—Si1	1.6037 (18)	O37—Si7	1.585 (2)
O4—Si21	1.5988 (18)	O37—Si17	1.584 (2)
O5—Si1	1.595 (2)	O38—Si14^v^	1.601 (2)
O5—Si13^iii^	1.602 (2)	O38—Si16	1.601 (2)
O6—Si6	1.5917 (18)	O39—Si8^vii^	1.603 (2)
O6—Si19	1.5941 (19)	O39—Si10	1.5975 (19)
O7—Si3	1.602 (2)	O40—Si12	1.590 (2)
O7—Si5	1.600 (2)	O40—Si23	1.600 (2)
O8—Si14	1.597 (2)	O41—Si11	1.595 (2)
O8—Si19	1.597 (2)	O41—Si12	1.602 (2)
O9—Si6	1.601 (2)	O42—Si5	1.594 (2)
O9—Si8	1.6047 (18)	O42—Si22	1.591 (2)
O10—Si3	1.606 (2)	O43—Si3	1.6036 (19)
O10—Si22^iv^	1.5980 (19)	O43—Si21^vi^	1.606 (2)
O11—Si11^v^	1.607 (2)	O44—Si19	1.591 (2)
O11—Si16	1.612 (2)	O44—Si22	1.604 (2)
O12—Si3	1.603 (2)	O45—Si15	1.601 (2)
O12—Si14	1.599 (2)	O45—Si18	1.596 (2)
O13—Si1	1.606 (2)	O46—Si5	1.5987 (19)
O13—Si18	1.597 (2)	O46—Si20	1.5923 (19)
O14—Si16	1.5932 (18)	O47—Si8	1.5998 (19)
O14—Si24	1.5950 (19)	O47—Si9	1.6031 (19)
O15—Si1	1.596 (2)	O48—Si12	1.598 (2)
O15—Si9	1.5915 (19)	O48—Si19	1.593 (2)
			
C1···O3	3.499 (1)	C6···O21^xii^	3.319 (1)
C2···O28	3.489 (1)	C6···O1	3.439 (1)
C3···O6	3.326 (1)	C6···O14^xii^	3.305 (1)
C3···O9	3.384 (1)	C10···O48^xii^	3.830 (1)
C3···O44	3.411 (1)	C11···O31^xii^	3.684 (1)
C4···O39	3.494 (1)	C11···O45^xii^	3.684 (1)
C5···O39	3.428 (1)	C13···O46^xii^	3.402 (1)
C5···O19^xii^	3.440 (1)	C14···O15^xii^	3.897 (1)
C5···O21^xii^	3.315 (1)	C14···O21^xii^	3.481 (1)
C5···O14^xii^	3.151 (1)		
			
C2—C1—C6	116.1 (5)	O25^xiii^—Si4—O17	108.92 (13)
C2—C1—C7	123.0 (5)	O25^xiii^—Si4—O28	108.06 (13)
C6—C1—C7	120.9 (5)	O25^xiii^—Si4—O34	110.41 (13)
C1—C2—H2	119.0	O28—Si4—O17	110.78 (12)
C3—C2—C1	121.9 (5)	O34—Si4—O17	109.26 (13)
C3—C2—H2	119.0	O34—Si4—O28	109.40 (13)
C2—C3—H3	119.2	O7—Si5—O1	110.63 (11)
C2—C3—C4	121.6 (5)	O42—Si5—O1	108.61 (11)
C4—C3—H3	119.2	O42—Si5—O7	110.40 (12)
C3—C4—C8	121.0 (5)	O42—Si5—O46	110.20 (12)
C5—C4—C3	116.4 (5)	O46—Si5—O1	107.41 (11)
C5—C4—C8	122.6 (5)	O46—Si5—O7	109.54 (12)
C4—C5—H5	118.6	O6—Si6—O9	108.10 (11)
C6—C5—C4	122.9 (4)	O6—Si6—O31	111.11 (12)
C6—C5—H5	118.6	O29^iv^—Si6—O6	109.85 (14)
C1—C6—H6	119.5	O29^iv^—Si6—O9	108.94 (13)
C5—C6—C1	121.1 (5)	O29^iv^—Si6—O31	109.30 (13)
C5—C6—H6	119.5	O31—Si6—O9	109.49 (13)
C1—C7—H7A	109.5	O2—Si7—O22^i^	109.63 (12)
C1—C7—H7B	109.5	O23^xiv^—Si7—O2	111.28 (11)
C1—C7—H7C	109.5	O23^xiv^—Si7—O22^i^	108.71 (13)
H7A—C7—H7B	109.5	O37—Si7—O2	107.77 (13)
H7A—C7—H7C	109.5	O37—Si7—O22^i^	110.17 (14)
H7B—C7—H7C	109.5	O37—Si7—O23^xiv^	109.27 (14)
C4—C8—H8A	109.5	O24—Si8—O9	111.36 (12)
C4—C8—H8B	109.5	O24—Si8—O39^xv^	110.03 (12)
C4—C8—H8C	109.5	O24—Si8—O47	108.54 (12)
H8A—C8—H8B	109.5	O39^xv^—Si8—O9	108.14 (10)
H8A—C8—H8C	109.5	O47—Si8—O9	108.53 (11)
H8B—C8—H8C	109.5	O47—Si8—O39^xv^	110.23 (11)
C10—C9—C15	121.9 (7)	O15—Si9—O19	109.91 (12)
C14—C9—C10	115.3 (7)	O15—Si9—O21^xv^	108.10 (11)
C14—C9—C15	122.7 (7)	O15—Si9—O47	109.19 (12)
C9—C10—H10	119.1	O19—Si9—O21^xv^	110.27 (13)
C9—C10—C11	121.7 (6)	O19—Si9—O47	110.70 (12)
C11—C10—H10	119.1	O21^xv^—Si9—O47	108.62 (11)
C10—C11—H11	119.0	O19^v^—Si10—O33	109.46 (14)
C12—C11—C10	122.1 (6)	O19^v^—Si10—O36	108.75 (13)
C12—C11—H11	119.0	O19^v^—Si10—O39	111.99 (12)
C11—C12—C13	115.7 (7)	O33—Si10—O36	110.71 (14)
C11—C12—C16	119.3 (7)	O39—Si10—O33	107.15 (11)
C13—C12—C16	125.0 (7)	O39—Si10—O36	108.79 (12)
C12—C13—H13	118.8	O2^viii^—Si11—O11^iv^	110.20 (10)
C14—C13—C12	122.5 (7)	O2^viii^—Si11—O35	108.42 (11)
C14—C13—H13	118.8	O35—Si11—O11^iv^	111.03 (11)
C9—C14—C13	122.7 (6)	O41—Si11—O2^viii^	110.36 (12)
C9—C14—H14	118.7	O41—Si11—O11^iv^	108.61 (12)
C13—C14—H14	118.7	O41—Si11—O35	108.20 (12)
C9—C15—H15A	109.5	O32—Si12—O41	109.01 (13)
C9—C15—H15B	109.5	O32—Si12—O48	108.01 (12)
C9—C15—H15C	109.5	O40—Si12—O32	112.09 (13)
H15A—C15—H15B	109.5	O40—Si12—O41	108.22 (12)
H15A—C15—H15C	109.5	O40—Si12—O48	108.22 (13)
H15B—C15—H15C	109.5	O48—Si12—O41	111.31 (13)
C12—C16—H16A	109.5	O5^xii^—Si13—O3^vi^	108.81 (11)
C12—C16—H16B	109.5	O24—Si13—O3^vi^	110.78 (11)
C12—C16—H16C	109.5	O24—Si13—O5^xii^	109.74 (13)
H16A—C16—H16B	109.5	O24—Si13—O26	108.86 (13)
H16A—C16—H16C	109.5	O26—Si13—O3^vi^	109.11 (12)
H16B—C16—H16C	109.5	O26—Si13—O5^xii^	109.53 (12)
Si16—O1—Si5	142.09 (13)	O8—Si14—O12	108.88 (13)
Si7—O2—Si11^i^	148.55 (16)	O8—Si14—O20	108.56 (13)
Si13^ii^—O3—Si23	140.93 (14)	O8—Si14—O38^iv^	110.01 (12)
Si21—O4—Si1	149.75 (15)	O12—Si14—O20	110.22 (12)
Si1—O5—Si13^iii^	151.47 (14)	O12—Si14—O38^iv^	109.66 (11)
Si6—O6—Si19	151.93 (15)	O38^iv^—Si14—O20	109.50 (12)
Si5—O7—Si3	155.15 (15)	O16—Si15—O20^ii^	111.26 (12)
Si14—O8—Si19	161.26 (17)	O16—Si15—O28	107.67 (13)
Si6—O9—Si8	137.81 (13)	O16—Si15—O45	110.36 (12)
Si22^iv^—O10—Si3	150.07 (13)	O20^ii^—Si15—O28	109.83 (12)
Si11^v^—O11—Si16	149.92 (13)	O45—Si15—O20^ii^	108.05 (12)
Si14—O12—Si3	161.42 (16)	O45—Si15—O28	109.68 (12)
Si18—O13—Si1	158.97 (16)	O1—Si16—O11	109.70 (11)
Si16—O14—Si24	152.08 (15)	O1—Si16—O38	109.65 (10)
Si9—O15—Si1	160.95 (15)	O14—Si16—O1	108.14 (11)
Si15—O16—Si23	159.03 (17)	O14—Si16—O11	110.51 (10)
Si21—O17—Si4	170.69 (17)	O14—Si16—O38	110.57 (11)
Si2—O18—Si24	145.42 (14)	O38—Si16—O11	108.27 (11)
Si10^iv^—O19—Si9	172.78 (19)	O26^v^—Si17—O30	109.69 (12)
Si14—O20—Si15^vi^	143.25 (16)	O36—Si17—O26^v^	110.72 (13)
Si24—O21—Si9^vii^	154.34 (15)	O36—Si17—O30	107.97 (12)
Si18—O22—Si7^viii^	148.09 (15)	O37—Si17—O26^v^	110.20 (13)
Si7^ix^—O23—Si24	151.12 (14)	O37—Si17—O30	108.20 (14)
Si13—O24—Si8	169.20 (16)	O37—Si17—O36	110.00 (15)
Si4^x^—O25—Si20	156.04 (16)	O13—Si18—O22	109.08 (12)
Si13—O26—Si17^iv^	170.40 (17)	O31—Si18—O13	110.19 (12)
Si2—O27—Si23^xi^	149.55 (16)	O31—Si18—O22	111.07 (13)
Si4—O28—Si15	157.69 (17)	O31—Si18—O45	108.00 (13)
Si2—O29—Si6^v^	171.5 (2)	O45—Si18—O13	110.01 (12)
Si20^xi^—O30—Si17	146.62 (16)	O45—Si18—O22	108.47 (12)
Si18—O31—Si6	166.55 (19)	O6—Si19—O8	109.66 (13)
Si12—O32—Si20	158.71 (17)	O44—Si19—O6	108.33 (12)
Si2—O33—Si10	153.45 (17)	O44—Si19—O8	111.34 (13)
Si4—O34—Si22^ii^	162.25 (18)	O44—Si19—O48	111.77 (13)
Si11—O35—Si21^x^	141.42 (14)	O48—Si19—O6	107.16 (12)
Si17—O36—Si10	147.11 (15)	O48—Si19—O8	108.48 (13)
Si17—O37—Si7	161.20 (19)	O30^xvi^—Si20—O25	109.45 (12)
Si16—O38—Si14^v^	150.11 (14)	O30^xvi^—Si20—O32	110.28 (13)
Si10—O39—Si8^vii^	145.09 (13)	O32—Si20—O25	110.13 (13)
Si12—O40—Si23	152.19 (17)	O46—Si20—O25	110.08 (12)
Si11—O41—Si12	151.79 (16)	O46—Si20—O30^xvi^	107.97 (11)
Si22—O42—Si5	169.20 (17)	O46—Si20—O32	108.90 (12)
Si3—O43—Si21^vi^	140.61 (14)	O4—Si21—O35^xiii^	110.57 (11)
Si19—O44—Si22	155.70 (16)	O4—Si21—O43^ii^	108.22 (11)
Si18—O45—Si15	146.81 (17)	O17—Si21—O4	109.97 (12)
Si20—O46—Si5	149.61 (14)	O17—Si21—O35^xiii^	108.78 (12)
Si8—O47—Si9	155.99 (15)	O17—Si21—O43^ii^	109.78 (12)
Si19—O48—Si12	153.85 (16)	O43^ii^—Si21—O35^xiii^	109.51 (11)
O4—Si1—O13	111.27 (12)	O10^v^—Si22—O34^vi^	108.28 (12)
O5—Si1—O4	108.75 (11)	O10^v^—Si22—O44	109.16 (11)
O5—Si1—O13	108.39 (11)	O34^vi^—Si22—O44	111.53 (13)
O5—Si1—O15	111.20 (12)	O42—Si22—O10^v^	109.69 (11)
O15—Si1—O4	107.18 (12)	O42—Si22—O34^vi^	110.08 (13)
O15—Si1—O13	110.06 (12)	O42—Si22—O44	108.08 (12)
O18—Si2—O27	108.40 (12)	O16—Si23—O3	108.58 (12)
O18—Si2—O29	111.21 (14)	O16—Si23—O27^xvi^	110.40 (13)
O18—Si2—O33	108.64 (12)	O16—Si23—O40	111.13 (12)
O27—Si2—O33	110.09 (13)	O27^xvi^—Si23—O3	110.29 (11)
O29—Si2—O27	109.65 (14)	O27^xvi^—Si23—O40	106.37 (12)
O29—Si2—O33	108.84 (14)	O40—Si23—O3	110.08 (12)
O7—Si3—O10	110.49 (11)	O14—Si24—O18	108.58 (11)
O7—Si3—O12	108.10 (12)	O14—Si24—O23	110.23 (11)
O7—Si3—O43	110.13 (11)	O18—Si24—O23	108.73 (13)
O12—Si3—O10	111.22 (11)	O21—Si24—O14	109.30 (11)
O12—Si3—O43	109.23 (12)	O21—Si24—O18	110.88 (12)
O43—Si3—O10	107.67 (10)	O21—Si24—O23	109.12 (13)
			
C1—C2—C3—C4	1.1 (8)	Si12—O40—Si23—O16	−87.2 (4)
C2—C1—C6—C5	1.0 (7)	Si12—O40—Si23—O27^xvi^	152.7 (3)
C2—C3—C4—C5	0.6 (7)	Si12—O41—Si11—O2^viii^	−93.5 (3)
C2—C3—C4—C8	−180.0 (5)	Si12—O41—Si11—O11^iv^	27.4 (4)
C3—C4—C5—C6	−1.5 (6)	Si12—O41—Si11—O35	148.0 (3)
C4—C5—C6—C1	0.7 (7)	Si12—O48—Si19—O6	−170.7 (4)
C6—C1—C2—C3	−1.8 (7)	Si12—O48—Si19—O8	71.0 (4)
C7—C1—C2—C3	177.7 (5)	Si12—O48—Si19—O44	−52.2 (5)
C7—C1—C6—C5	−178.5 (5)	Si13^ii^—O3—Si23—O16	−171.0 (2)
C8—C4—C5—C6	179.1 (5)	Si13^ii^—O3—Si23—O27^xvi^	−49.9 (2)
C9—C10—C11—C12	−1.4 (9)	Si13^ii^—O3—Si23—O40	67.2 (2)
C10—C9—C14—C13	0.0 (9)	Si13^iii^—O5—Si1—O4	−139.4 (3)
C10—C11—C12—C13	1.5 (8)	Si13^iii^—O5—Si1—O13	−18.3 (3)
C10—C11—C12—C16	−179.3 (6)	Si13^iii^—O5—Si1—O15	102.8 (3)
C11—C12—C13—C14	−0.9 (8)	Si13—O24—Si8—O9	−10.5 (10)
C12—C13—C14—C9	0.2 (9)	Si13—O24—Si8—O39^xv^	109.4 (10)
C14—C9—C10—C11	0.6 (8)	Si13—O24—Si8—O47	−129.9 (10)
C15—C9—C10—C11	179.7 (6)	Si14—O8—Si19—O6	143.8 (5)
C15—C9—C14—C13	−179.2 (6)	Si14—O8—Si19—O44	23.9 (6)
C16—C12—C13—C14	179.9 (6)	Si14—O8—Si19—O48	−99.4 (6)
Si1—O4—Si21—O17	34.4 (3)	Si14—O12—Si3—O7	−136.9 (5)
Si1—O4—Si21—O35^xiii^	−85.8 (3)	Si14—O12—Si3—O10	−15.4 (6)
Si1—O4—Si21—O43^ii^	154.3 (3)	Si14—O12—Si3—O43	103.3 (5)
Si1—O13—Si18—O22	180.0 (4)	Si14^v^—O38—Si16—O1	−157.8 (3)
Si1—O13—Si18—O31	−57.8 (5)	Si14^v^—O38—Si16—O11	−38.1 (3)
Si1—O13—Si18—O45	61.1 (5)	Si14^v^—O38—Si16—O14	83.1 (3)
Si1—O15—Si9—O19	34.0 (5)	Si15—O16—Si23—O3	173.4 (5)
Si1—O15—Si9—O21^xv^	154.4 (5)	Si15—O16—Si23—O27^xvi^	52.4 (5)
Si1—O15—Si9—O47	−87.6 (5)	Si15—O16—Si23—O40	−65.4 (5)
Si2—O18—Si24—O14	−159.6 (3)	Si15^vi^—O20—Si14—O8	54.9 (3)
Si2—O18—Si24—O21	80.3 (3)	Si15^vi^—O20—Si14—O12	−64.3 (3)
Si2—O18—Si24—O23	−39.7 (3)	Si15^vi^—O20—Si14—O38^iv^	175.0 (2)
Si2—O33—Si10—O19^v^	−61.0 (4)	Si15—O28—Si4—O17	5.7 (5)
Si2—O33—Si10—O36	58.9 (4)	Si15—O28—Si4—O25^xiii^	124.9 (4)
Si2—O33—Si10—O39	177.4 (4)	Si15—O28—Si4—O34	−114.9 (4)
Si3—O7—Si5—O1	−126.7 (3)	Si15—O45—Si18—O13	85.9 (3)
Si3—O7—Si5—O42	−6.5 (4)	Si15—O45—Si18—O22	−33.3 (3)
Si3—O7—Si5—O46	115.0 (4)	Si15—O45—Si18—O31	−153.8 (3)
Si3—O12—Si14—O8	139.6 (5)	Si16—O1—Si5—O7	96.5 (2)
Si3—O12—Si14—O20	−101.5 (5)	Si16—O1—Si5—O42	−24.8 (3)
Si3—O12—Si14—O38^iv^	19.2 (6)	Si16—O1—Si5—O46	−144.0 (2)
Si4^x^—O25—Si20—O30^xvi^	164.7 (4)	Si16—O14—Si24—O18	59.2 (3)
Si4^x^—O25—Si20—O32	43.3 (4)	Si16—O14—Si24—O21	−179.7 (3)
Si4^x^—O25—Si20—O46	−76.7 (4)	Si16—O14—Si24—O23	−59.8 (3)
Si4—O28—Si15—O16	−179.5 (4)	Si17—O36—Si10—O19^v^	34.2 (4)
Si4—O28—Si15—O20^ii^	59.2 (5)	Si17—O36—Si10—O33	−86.1 (4)
Si4—O28—Si15—O45	−59.4 (5)	Si17—O36—Si10—O39	156.4 (3)
Si5—O1—Si16—O11	−51.5 (2)	Si17—O37—Si7—O2	−156.0 (6)
Si5—O1—Si16—O14	−172.1 (2)	Si17—O37—Si7—O22^i^	84.4 (7)
Si5—O1—Si16—O38	67.3 (2)	Si17—O37—Si7—O23^xiv^	−35.0 (7)
Si5—O7—Si3—O10	−145.9 (3)	Si18—O13—Si1—O4	−66.1 (5)
Si5—O7—Si3—O12	−24.0 (4)	Si18—O13—Si1—O5	174.4 (4)
Si5—O7—Si3—O43	95.2 (4)	Si18—O13—Si1—O15	52.6 (5)
Si5—O42—Si22—O10^v^	−159.7 (9)	Si18—O31—Si6—O6	−85.1 (7)
Si5—O42—Si22—O34^vi^	−40.7 (9)	Si18—O31—Si6—O9	155.6 (7)
Si5—O42—Si22—O44	81.4 (9)	Si18—O31—Si6—O29^iv^	36.3 (8)
Si5—O46—Si20—O25	92.3 (3)	Si18—O45—Si15—O16	76.3 (3)
Si5—O46—Si20—O30^xvi^	−148.3 (3)	Si18—O45—Si15—O20^ii^	−161.9 (3)
Si5—O46—Si20—O32	−28.5 (3)	Si18—O45—Si15—O28	−42.2 (3)
Si6—O6—Si19—O8	63.9 (4)	Si19—O6—Si6—O9	175.5 (3)
Si6—O6—Si19—O44	−174.4 (3)	Si19—O6—Si6—O29^iv^	−65.7 (4)
Si6—O6—Si19—O48	−53.6 (4)	Si19—O6—Si6—O31	55.3 (4)
Si6—O9—Si8—O24	−72.4 (2)	Si19—O8—Si14—O12	11.0 (6)
Si6—O9—Si8—O39^xv^	166.6 (2)	Si19—O8—Si14—O20	−109.0 (6)
Si6—O9—Si8—O47	47.0 (2)	Si19—O8—Si14—O38^iv^	131.2 (5)
Si6—O31—Si18—O13	−93.5 (7)	Si19—O44—Si22—O10^v^	179.1 (4)
Si6—O31—Si18—O22	27.5 (8)	Si19—O44—Si22—O34^vi^	59.5 (4)
Si6—O31—Si18—O45	146.3 (7)	Si19—O44—Si22—O42	−61.6 (4)
Si7^viii^—O22—Si18—O13	39.6 (4)	Si19—O48—Si12—O32	9.7 (5)
Si7^viii^—O22—Si18—O31	−82.1 (3)	Si19—O48—Si12—O40	131.2 (4)
Si7^viii^—O22—Si18—O45	159.4 (3)	Si19—O48—Si12—O41	−110.0 (4)
Si7^ix^—O23—Si24—O14	−11.7 (4)	Si20^xi^—O30—Si17—O26^v^	−33.1 (3)
Si7^ix^—O23—Si24—O18	−130.6 (4)	Si20^xi^—O30—Si17—O36	−153.9 (3)
Si7^ix^—O23—Si24—O21	108.3 (4)	Si20^xi^—O30—Si17—O37	87.1 (3)
Si7—O37—Si17—O26^v^	−26.9 (7)	Si20—O32—Si12—O40	53.8 (5)
Si7—O37—Si17—O30	−146.8 (6)	Si20—O32—Si12—O41	−66.0 (5)
Si7—O37—Si17—O36	95.5 (6)	Si20—O32—Si12—O48	172.9 (5)
Si8—O9—Si6—O6	154.8 (2)	Si20—O46—Si5—O1	−179.2 (3)
Si8—O9—Si6—O29^iv^	35.5 (3)	Si20—O46—Si5—O7	−59.0 (3)
Si8—O9—Si6—O31	−84.0 (2)	Si20—O46—Si5—O42	62.7 (3)
Si8—O24—Si13—O3^vi^	9.0 (10)	Si21—O4—Si1—O5	52.5 (3)
Si8—O24—Si13—O5^xii^	−111.2 (10)	Si21—O4—Si1—O13	−66.8 (3)
Si8—O24—Si13—O26	129.0 (10)	Si21—O4—Si1—O15	172.8 (3)
Si8^vii^—O39—Si10—O19^v^	−92.1 (3)	Si21^x^—O35—Si11—O2^viii^	179.5 (2)
Si8^vii^—O39—Si10—O33	28.0 (3)	Si21^x^—O35—Si11—O11^iv^	58.3 (2)
Si8^vii^—O39—Si10—O36	147.7 (2)	Si21^x^—O35—Si11—O41	−60.8 (2)
Si8—O47—Si9—O15	138.2 (3)	Si21^vi^—O43—Si3—O7	−60.1 (2)
Si8—O47—Si9—O19	17.1 (4)	Si21^vi^—O43—Si3—O10	179.3 (2)
Si8—O47—Si9—O21^xv^	−104.1 (4)	Si21^vi^—O43—Si3—O12	58.4 (2)
Si9—O15—Si1—O4	165.1 (5)	Si22^iv^—O10—Si3—O7	46.1 (3)
Si9—O15—Si1—O5	−76.2 (5)	Si22^iv^—O10—Si3—O12	−74.0 (3)
Si9—O15—Si1—O13	43.9 (5)	Si22^iv^—O10—Si3—O43	166.4 (3)
Si9^vii^—O21—Si24—O14	141.1 (3)	Si22^ii^—O34—Si4—O17	−56.2 (6)
Si9^vii^—O21—Si24—O18	−99.2 (4)	Si22^ii^—O34—Si4—O25^xiii^	−176.0 (5)
Si9^vii^—O21—Si24—O23	20.5 (4)	Si22^ii^—O34—Si4—O28	65.2 (6)
Si9—O47—Si8—O9	−100.6 (4)	Si22—O42—Si5—O1	117.1 (9)
Si9—O47—Si8—O24	20.6 (4)	Si22—O42—Si5—O7	−4.4 (9)
Si9—O47—Si8—O39^xv^	141.2 (3)	Si22—O42—Si5—O46	−125.5 (9)
Si10—O33—Si2—O18	−177.8 (4)	Si22—O44—Si19—O6	−133.9 (4)
Si10—O33—Si2—O27	−59.2 (4)	Si22—O44—Si19—O8	−13.3 (5)
Si10—O33—Si2—O29	61.0 (4)	Si22—O44—Si19—O48	108.2 (4)
Si10—O36—Si17—O26^v^	36.0 (4)	Si23—O16—Si15—O20^ii^	−52.8 (5)
Si10—O36—Si17—O30	156.1 (3)	Si23—O16—Si15—O28	−173.2 (5)
Si10—O36—Si17—O37	−86.0 (4)	Si23—O16—Si15—O45	67.1 (5)
Si11^i^—O2—Si7—O22^i^	−56.5 (3)	Si23^xi^—O27—Si2—O18	−123.4 (3)
Si11^i^—O2—Si7—O23^xiv^	63.8 (3)	Si23^xi^—O27—Si2—O29	−1.8 (4)
Si11^i^—O2—Si7—O37	−176.4 (3)	Si23^xi^—O27—Si2—O33	117.9 (3)
Si11^v^—O11—Si16—O1	−95.1 (3)	Si23—O40—Si12—O32	−108.7 (3)
Si11^v^—O11—Si16—O14	24.1 (3)	Si23—O40—Si12—O41	11.5 (4)
Si11^v^—O11—Si16—O38	145.3 (3)	Si23—O40—Si12—O48	132.3 (3)
Si11—O41—Si12—O32	−92.7 (4)	Si24—O14—Si16—O1	176.6 (3)
Si11—O41—Si12—O40	145.1 (3)	Si24—O14—Si16—O11	56.5 (3)
Si11—O41—Si12—O48	26.3 (4)	Si24—O14—Si16—O38	−63.3 (3)
Si12—O32—Si20—O25	71.4 (5)	Si24—O18—Si2—O27	−160.7 (3)
Si12—O32—Si20—O30^xvi^	−49.5 (5)	Si24—O18—Si2—O29	78.7 (3)
Si12—O32—Si20—O46	−167.8 (5)	Si24—O18—Si2—O33	−41.1 (3)
Si12—O40—Si23—O3	33.2 (4)		

Symmetry codes: (i) *x*, *y*+1, *z*+1; (ii) *x*+1/2, −*y*+1/2, −*z*+1; (iii) −*x*+1/2, −*y*, *z*−1/2; (iv) −*x*+1/2, −*y*+1, *z*−1/2; (v) −*x*+1/2, −*y*+1, *z*+1/2; (vi) *x*−1/2, −*y*+1/2, −*z*+1; (vii) *x*, *y*+1, *z*; (viii) *x*, *y*−1, *z*−1; (ix) −*x*+1/2, −*y*+2, *z*−1/2; (x) −*x*+1, *y*+1/2, −*z*+1/2; (xi) −*x*+1, *y*+1/2, −*z*+3/2; (xii) −*x*+1/2, −*y*, *z*+1/2; (xiii) −*x*+1, *y*−1/2, −*z*+1/2; (xiv) −*x*+1/2, −*y*+2, *z*+1/2; (xv) *x*, *y*−1, *z*; (xvi) −*x*+1, *y*−1/2, −*z*+3/2.
